# Reactive glia promote development of CD103^+^CD69^+^ CD8^+^ T‐cells through programmed cell death‐ligand 1 (PD‐L1)

**DOI:** 10.1002/iid3.221

**Published:** 2018-03-30

**Authors:** Sujata Prasad, Shuxian Hu, Wen S. Sheng, Priyanka Chauhan, James R. Lokensgard

**Affiliations:** ^1^ Neurovirology Laboratory Department of Medicine University of Minnesota Minnesota USA

**Keywords:** bT_RM_, central nervous system, CD103, glial cells, PD‐L1

## Abstract

**Introduction:**

Previous work from our laboratory has demonstrated in vivo persistence of CD103^+^CD69^+^ brain resident memory CD8^+^ T‐cells (bT_RM_) following viral infection, and that the PD‐1: PD‐L1 pathway promotes development of these T_RM_ cells within the brain. Although glial cells express low basal levels of PD‐L1, its expression is upregulated upon IFN‐γ‐treatment, and they have been shown to modulate antiviral T‐cell effector responses through the PD‐1: PD‐L1 pathway.

**Methods:**

We performed flow cytometric analysis of cells from co‐cultures of mixed glia and CD8^+^ T‐cells obtained from wild type mice to investigate the role of glial cells in the development of bT_RM_.

**Results:**

In this study, we show that interactions between reactive glia and anti‐CD3 Ab‐stimulated CD8^+^ T‐cells promote development of CD103^+^CD69^+^ CD8^+^ T‐cells through engagement of the PD‐1: PD‐L1 pathway. These studies used co‐cultures of primary murine glial cells obtained from WT animals along with CD8^+^ T‐cells obtained from either WT or PD‐1 KO mice. We found that αCD3 Ab‐stimulated CD8^+^ T‐cells from WT animals increased expression of CD103 and CD69 when co‐cultured with primary murine glial cells. In contrast, significantly reduced expression of CD103 and CD69 was observed using CD8^+^ T‐cells from PD‐1 KO mice. We also observed that reactive glia promoted high levels of CD127, a marker of memory precursor effector cells (MPEC), on CD69^+^ CD8^+^ T‐cells, which promotes development of T_RM_ cells. Interestingly, results obtained using T‐cells from PD‐1 KO animals showed significantly reduced expression of CD127 on CD69^+^ CD8^+^ cells. Additionally, blocking of glial PD‐L1 resulted in decreased expression of CD103, along with reduced CD127 on CD69^+^ CD8^+^ T‐cells.

**Conclusions:**

Taken together, these results demonstrate a role for activated glia in promoting development of bT_RM_ through the PD‐1: PD‐L1 pathway.

## Introduction

Microglial cells are the major immune component of the central nervous system (CNS) and are key mediators of neuroinflammatory processes. Being resident innate immune cells, they provide the first line of defense against CNS insult and acute inflammation. Activation of these glial cells leads to their accumulation at sites of injury or inflammation. Potent immune responses are critical to enhance pathogen clearance, but prolonged responses following neuroinflammation can be detrimental to brain tissue. Both microglial cells and astrocytes upregulate MHC class I and II, as well as programmed death ligand (PD‐L)‐1 upon activation, suggesting interaction with CNS‐infiltrating lymphocytes 1, 2. Although critical in pathogen clearance, growing evidence suggests that glial cells also function to modulate the responses of brain‐infiltrating immune cells through proinflammatory or anti‐inflammatory cytokine production 3, 4. However, involvement of reactive glia in the modulation of cells of the adaptive immune response is largely unknown.

Engagement of PD‐1 (CD279) to its ligands PD‐L1 and PD‐L2 plays a critical role in eliciting inhibitory second signals that regulate the balance between T‐cell activation and tolerance. Upregulation of PD‐1 and PD‐L1 following infection and inflammation has drawn much attention over the past few decades. Previous studies demonstrate that IFN‐γ stimulation results in enhanced expression of PD‐L1 on glial cells, whereas antigen experienced CD8^+^ T‐cells express PD‐1, the receptor of PD‐L1 1, 5, 6, 7. Extensive studies from murine models illustrate the immunoregulatory role of microglia during chronic persistent neuroinflammation. A number of studies from post‐encephalitic brains suggests that glial cells inhibit CD8^+^ T‐cell activation through the PD‐1: PD‐L1 pathway 1, 2, 5, 8. Additionally, blocking the interaction of PD‐1: PD‐L1 between CD8^+^ T‐cells and either microglia or astrocytes, resulted in increased IFN‐γ and IL‐2 production 1.

To provide protection against viral infection, tissue‐infiltrating CD8^+^ T‐cells differentiate into several distinct subsets 9, 10, 11, 12. Tissue resident memory (T_RM_) CD8^+^ T‐cells are one of these unique subsets that develop following resolution of primary infection to impart long‐term immunity against re‐infection 13, 14, 15. In many tissues, persistence of these T_RM_ is mediated by the specific adhesion molecule CD103 (i.e., integrin αeβ7) 15, 16. Bona fide T_RMs_ have also been reported to express CD69, which interferes with sphingosine 1‐phosphate receptor 1 (S1P1) on effector T‐cells and prevents tissue egress 17. Additionally, surface expression of CD103 for T_RM_ maintenance varies depending on the location and type of tissue 18. Long‐lived CD103^−^ T_RMs_ have been well‐studied in secondary lymphoid organs, female reproductive tract, and gut 14, 19, 20.

Because of the enhanced protective immunity afforded by T_RM_ cells, there has been considerable progress in understanding their formation in various tissues, but how antigen specific CD8^+^ T‐cells acquire a resident‐memory phenotype within the CNS microenvironment following viral infection remains to be elucidated. In some tissues, persistence of viral antigen is thought to influence retention of T_RM_, while other studies show expression of CD103 and CD69 remain elevated following viral infection in the absence of detectable viral antigen. These studies indicate that continued presence of virus is not necessary to express these markers. Still other studies demonstrate upregulation of certain receptors, which in turn may retain T_RMs_ 21, 22, 23. Previous findings from our laboratory reported that CD103^+^ CD69^+^ CD8^+^ T‐cells persist within the brain of murine cytomegalovirus (MCMV)‐infected animals. We also reported that PD‐1: PD‐L1 signaling altered expression CD103 and CD69 10. There is also evidence that PD‐L1 expression on microglia regulates the differentiation of Th1 cells via nitric oxide, suggesting a role for these cells in regulating T‐cells 2. In vitro studies demonstrate that antigen‐pulsed macrophages and dendritic cells injected into mice stimulate CD8^+^ T‐cell to proliferate, show effector function, and differentiate into long‐lived memory cells 24. However, the glial cell: T‐cell interactions which result in generation of long‐term antigen specific bT_RM_ remains to be determined.

Extensive murine studies over the past few years have defined expression patterns of the T‐box transcription factors T‐bet (T‐box expressed in T‐cell) and Eomes (Eomesodermin), which regulate development of short‐lived effector T‐cells (SLEC) and memory precursor effector cells (MPEC) 25, 26, 27, 28. Effector CD8 T‐cells are characterized by the high expression of T‐bet whereas low levels promote the development of memory cells 29, 30, 31. It is likely that these transcription factors are also relevant in bT_RM_ development.

To better understand how the brain microenvironment shapes infiltrating CD8^+^ T‐cells to generate bT_RM_, we evaluated development of CD103 and CD69 expression on CD8^+^ T‐cells in the presence and absence of reactive glia.

## Materials and Methods

### Ethical statement

This study was carried out in strict accordance with recommendations in the Guide for the Care and Use of Laboratory Animals of the National Institutes of Health. The protocol was approved by the Institutional Animal Care and Use Committee (Protocol Number: 1701‐34539A) of the University of Minnesota. All surgery was performed under Ketamine/Xylazine anesthesia and all efforts were made to minimize suffering.

### Virus and animals

RM461, a MCMV expressing Escherichia coli β‐galactosidase under the control of the human ie1/ie2 promoter/enhancer 32 was kindly provided by Edward S. Mocarski. The virus was maintained by passage in weanling female Balb/c mice. Salivary gland‐passed virus was then grown in NIH 3T3 cells for 2 passages, which minimized any carry‐over of salivary gland tissue. Infected 3T3 cultures were harvested at 80–100% cytopathic effect and subjected to three freeze–thaw cycles. Cellular debris was removed by centrifugation (1000×*g*) at 4°C, and the virus was pelleted through a 35% sucrose cushion (in Tris‐buffered saline [50 mM Tris–HCl, 150 mM NaCl, pH 7.4]) at 23,000×*g* for 2 h at 4°C. The pellet was suspended in Tris buffered saline containing 10% heat‐inactivated fetal bovine serum (FBS). Viral stock titers were determined on 3T3 cells as 50% tissue culture infective doses (TCID_50_) per milliliter. Six to eight weeks old C57BL/6 mice were obtained from Charles River Laboratories (Wilmington, MA), while PD‐L1 KO and PD‐1 KO animals were kindly provided by Arlene Sharpe (Harvard University) and Sing Sing Way (Cincinnati Children's Hospital, Cincinnati, OH), respectively.

### Intracerebroventricular infection of mice

Infection of mice with MCMV was performed as previously described 33. Briefly, female mice (6–8 week old) were anesthetized using a combination of Ketamine and Xylazine (100 mg and 10 mg/kg body weight, respectively) and immobilized on a small animal stereotactic instrument equipped with a Cunningham mouse adapter (Stoelting Co., Wood Dale, IL). The skin and underlying connective tissue were reflected to expose reference sutures (sagittal and coronal) on the skull. The sagittal plane was adjusted such that bregma and lambda were positioned at the same coordinates on the vertical plane. Virulent, salivary gland‐passaged MCMV RM461 (1 × 10^5^ TCID50 units in 10 µl), was injected into the right lateral ventricle at 0.9 mm lateral, 0.5 mm caudal, and 3.0 mm ventral to bregma using a Hamilton syringe (10 µl) fitted to a 27 G needle. The injection was delivered over a period of 3–5 min. The opening in the skull was sealed with bone wax and the skin was closed using 4–0 silk sutures with a FS‐2 needle (Ethicon, Somerville NJ).

### Brain leukocyte isolation and flow cytometry analysis

Brain mononuclear cells were isolated from MCMV‐infected C57BL/6 WT and PD‐L1 KO mice, using a previously described procedure with minor modifications 34, 35, 36, 37. In brief, whole brain tissues were harvested (*n* = 3–4 animals/group/experiment), and minced finely using a scalpel in RPMI 1640 (2 g/L D‐glucose and 10 mM HEPES) and digested in 0.0625% trypsin (in Ca/Mg‐free HBSS) at room temperature for 20 min. Single cell preparations of infected brains were resuspended in 30% Percoll (Sigma–Aldrich, St. Louis, MO, USA) and banded on a 70% Percoll cushion at 900 × g for 30 min at 15°C. Brain leukocytes obtained from the 30–70% Percoll interface were collected. Following preparation of single cell suspensions, cells were treated with Fc block (anti‐CD32/CD16 in the form of 2.4G2 hybridoma culture supernatant with 2% normal rat and 2% normal mouse serum) to inhibit nonspecific Ab binding. Cells were then counted using the trypan blue dye exclusion method, and 1 × 10^6^ cells were subsequently stained with anti‐mouse immune cell surface markers for 15–20 min at 4°C (anti‐CD45‐PE‐Cy5, anti‐KLRG1‐PE‐Cy7, anti‐CD103‐FITC, anti‐CD69‐e‐F 450, (eBioscience, San Diego, CA) and anti‐CD8‐BV‐510 (Biolegend, San Diego, CA). For intracellular staining, cells were stained for 30 min with anti‐IFN‐γ ef450 obtained from eBioscience. Control isotype Abs were used for all fluorochrome combinations to assess nonspecific Ab binding. For tetramer staining, an MHC class I (H‐2D^b^) tetramer containing the M45 (HGIRNASFI) T‐cell epitope 38 was obtained from the NIH Tetramer Core Facility at Emory University and used for evaluation of viral antigen‐specific CD8^+^ T‐cell responses. Live leukocytes were gated using forward scatter and side scatter parameters on a BD FACS Canto flow cytometer and LSRII H4760 (BD Biosciences, San Jose, CA). Data were analyzed using FlowJo software (FlowJo, Ashland, OR).

### Primary mixed glial cell culture

Primary mixed glial cell cultures were established after dispersion of murine neonatal (<24 h of birth) cerebral cortices with trypsin (0.25%) for 30 min as previously described 1. Cells (5 × 10^4^/300 µl) were plated into wells of 24‐well plates with DMEM containing 5% heat—inactivated FBS, penicillin (100 U/ml), and streptomycin (100 µg/ml), and were then incubated at 37°C with 10% CO_2_. On the following day, the culture medium was replaced with 5% FBS‐DMEM. The culture medium was changed again 24 h later and every 3 d thereafter. On day 9, there were approximately 75% astrocytes, as determined by glial fibrillary acid protein staining (DAKO, Carpinteria, CA), and 25% microglial cells, as determined by anti‐Iba‐1 Ab (Wako Chemicals, Richmond,VA).

### CD8: Glial cell co‐culture

CD8^+^ T‐cells were isolated using the MagCellect Mouse CD8^+^ T Cell Isolation Kit (R&D Systems, Minneapolis, MN, USA) from the spleens of naive C57BL/6 and PD‐1 KO mice. Purified CD8^+^ T‐cells were placed into culture and stimulated with anti‐CD3 (2 µg/ml) Ab for 1 h prior to transfer onto mixed glial cell culture. CD8^+^ T‐cells were added at a 10:1 CD8: glial cell ratio. Neutralization of PD‐1 and its ligand was performed by treating glial cells with anti‐PD‐1 (J43 clone; eBiosciences, San Diego, CA, USA), anti‐PD‐L1 (M1H5 clone; eBiosciences), anti‐PD‐L2 (TY25 clone; eBiosciences), or IgG2a for 2 h prior to the addition of anti‐CD3‐activated CD8^+^ T‐cells. Cells were collected 48 h after the addition of T‐cells and stained for 15–20 min at 4°C for surface markers anti‐CD45‐PE‐Cy5, anti‐KLRG1‐PE‐Cy7, anti‐CD103‐FITC (clone 2E7), anti‐CD127‐APC, anti‐CD69‐e‐F 450, (eBioscience, San Diego CA), and anti‐CD8‐BV‐510 from (Biolegend). For intracellular staining, cells were stained for 30 min with anti‐IFN‐γ ef450, T‐bet‐PE (eBioscience), and EOMES‐PE‐Cy7 (Invitrogen, Carlsbad, CA). Control isotype Abs were used to assess nonspecific Ab binding.

### In situ tetramer staining combined with immunohistochemistry

In situ tetramer staining combined with immunohistochemistry was performed as described previously 39, 40. For sectioning, fresh tissues were embedded in 4% low melt agarose, cut into 200 micron thick sections, and incubated with FITC‐conjugated MHC class 1 tetramer at a concentration of 0.5 µg/ml, rat anti‐mouse CD8 (eBiosciences, diluted to 10 µg/ml) Abs in 1 ml of cold phosphate buffered saline containing 100 mg/ml heparin (PBS‐H) with 2% normal goat serum at 4°C overnight. Brain sections were then washed with chilled PBS‐H, fixed with 4% paraformaldehyde for 2 h at room temperature, and again washed with PBS‐H. For co‐labeling epitopes, prior to secondary incubation, tissues were boiled three times in 0.01 M Urea to expose epitopes, then permeabilized and blocked with PBS‐H containing 0.3% triton X–100 and 2% normal goat serum for 1 h. For the secondary incubation, rabbit anti‐FITC Abs (Invitrogen) diluted 1∶5000 in blocking solution were used at 4°C on a rocking platform overnight. Brain sections were then washed with PBS‐H and incubated with CY3‐conjugated goat anti‐rabbit Abs diluted 1∶5000, and Alexa 488–conjugated donkey anti‐rat Ab (Jackson ImmunoResearch, 1:400) in blocking solution for 24 h, followed by washing and post‐fixation with 4% paraformaldehyde for 1 h, and mounted on slides with warmed glycerol gelatin (Sigma) containing 4 mg/ml *n*‐propyl gallate.

### Statistical analysis

For comparing groups, two‐tailed unpaired Student's *T*‐test for samples was applied, *p* values ≤0.05 were considered significant.

## Results

### Antigen‐specific CD8^+^CD103^+^ T‐cells persisted within the brain following viral infection

**Figure 1:**
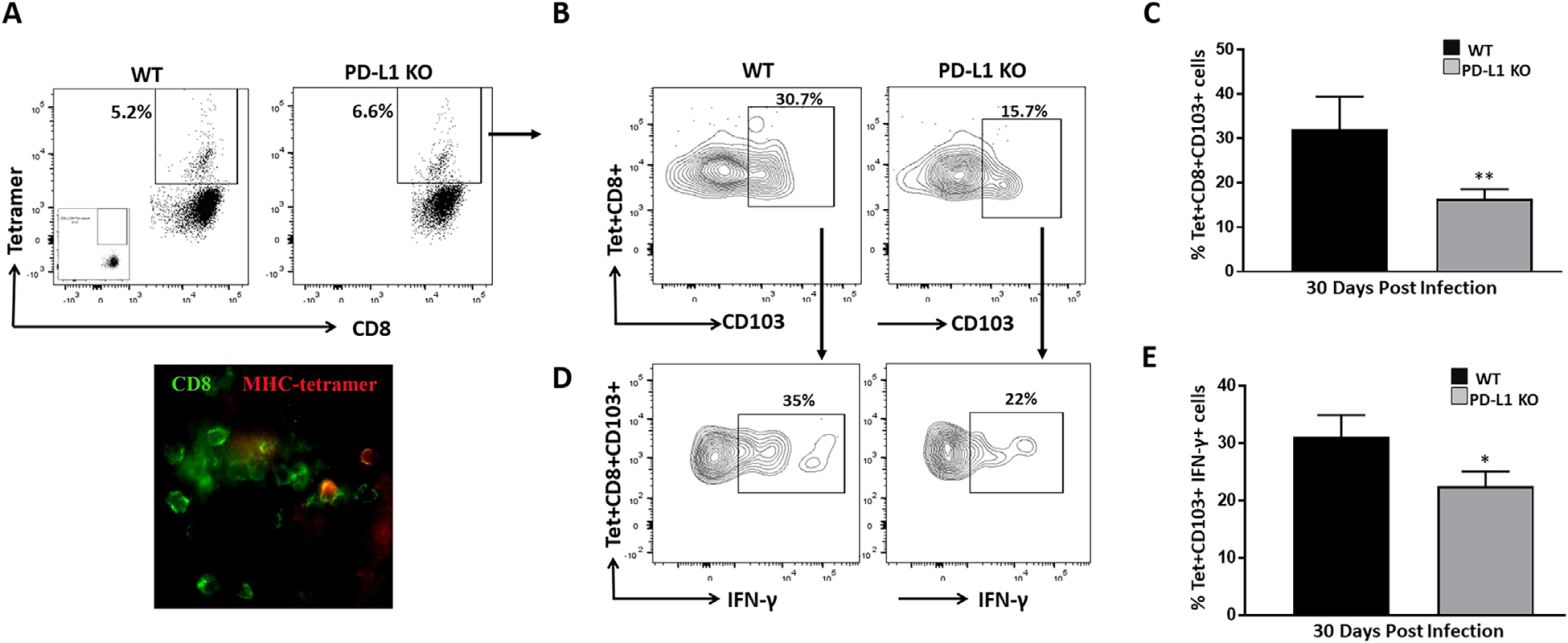
Antigen‐specific CD8^+^CD103^+^ T‐cells persisted within the brain following viral infection. Brain mononuclear cells obtained from MCMV‐infected WT and PD‐L1 KO animals were collected at d 30 and analyzed using an MHC class I M45 tetramer‐PE for MCMV‐specific T‐cells by flow cytometry. C57BL/6 mice were perfused and cryosectioned for immunohistochemistry. (A) Representative contour plots (upper panel) and image of immunostained brain sections (lower panel) for MCMV tetramer‐specific CD8^+^ T‐cells from WT animals at 30dpi. (B) CNS‐derived lymphocytes were gated on tetramer‐specific CD8^+^ T‐cells and representative contour plots show antigen‐specific brain resident memory T‐cells (bT_RM_) cells in WT and PD‐L1 KO mice at 30dpi. (C) Pooled data present frequency (mean ± SD) of tetramer‐specific CD8^+^ CD103^+^ T‐cells within infected brains of WT and PD‐L1 KO animals at the indicated time point from two independent experiments using 2–3 animals per group. **p < 0.01 WT versus PD‐L1 KO. (D) Flow cytometric contour plots represent IFN‐γ production by antigen‐specific bT_RM_ cells from WT and PD‐L1 KO mice at 30 dpi. (E) Pooled data obtained from two independent experiments present frequency (mean ± SD) of IFN‐γ production by tetramer‐specific CD8^+^ CD103^+^ T‐cells within the infected brains of WT and PD‐L1 KO animals at the indicated time point. *p < 0.05 WT versus PD‐L1 KO.

In our previous study, we used a well‐established mouse model of MCMV brain infection to demonstrate a role for the PD‐1: PD‐L1 pathway in development of CD103^+^CD69^+^ CD8^+^ bT_RM_ populations in vivo following acute viral infection 10. Here, we followed‐up on those findings by first demonstrating that some of the bT_RM_ were specific for a previously identified viral T‐cell epitope 38. We infected wild‐type (WT) C57BL/6 and PD‐L1 KO mice intracerebroventricularly with MCMV and evaluated expression of CD103 (marker for T_RM_) on antigen‐specific CD8^+^ T‐cells at 30 days post‐infection (dpi). Flow cytometric data revealed that 4.4 ± 1.2% and 5.0 ± 1.1% of the CD8^+^ T‐cells within the brain were specific for the MCMV epitope M45 tetramer at 30 dpi among WT and PD‐L1 KO animals, respectively (Fig. 1A). In addition, using immunohistochemical staining, we further confirmed that sections of infected brain at 30 dpi contained tetramer‐specific CD8^+^ T‐cells (Fig. 1A, lower panel). Development of a memory phenotype on antigen‐specific CD8^+^ T‐cells was further evaluated by assessing expression of CD103. In these experiments, we observed significantly higher expression of CD103 on antigen‐specific cells among WT animals (32 ± 7.3%) than in PD‐L1 KO mice (16.3 ± 2.1%), (Fig. 1B, C). We next assessed the functional capacity of CD103^+^ CD8^+^ T‐cells. Antigen‐specific CD8^+^ CD103^+^ T‐cells revealed production of IFN‐γ following ex vivo re‐stimulation with M45 peptide. In these studies, 31 ± 3.8% of the antigen‐specific CD8^+^ CD103^+^ T‐cells from WT animals produced IFN‐γ. Correspondingly, reduced IFN‐γ production was noted by antigen specific CD8^+^ CD103^+^ T‐cells from PD‐L1 KO animals 22.4 ± 2.5% (Fig. 1D, E).

### Increased expression of CD103 as well as co‐expression of CD103 and CD69 on CD8^+^ T‐cells in the presence of glia

**Figure 2:**
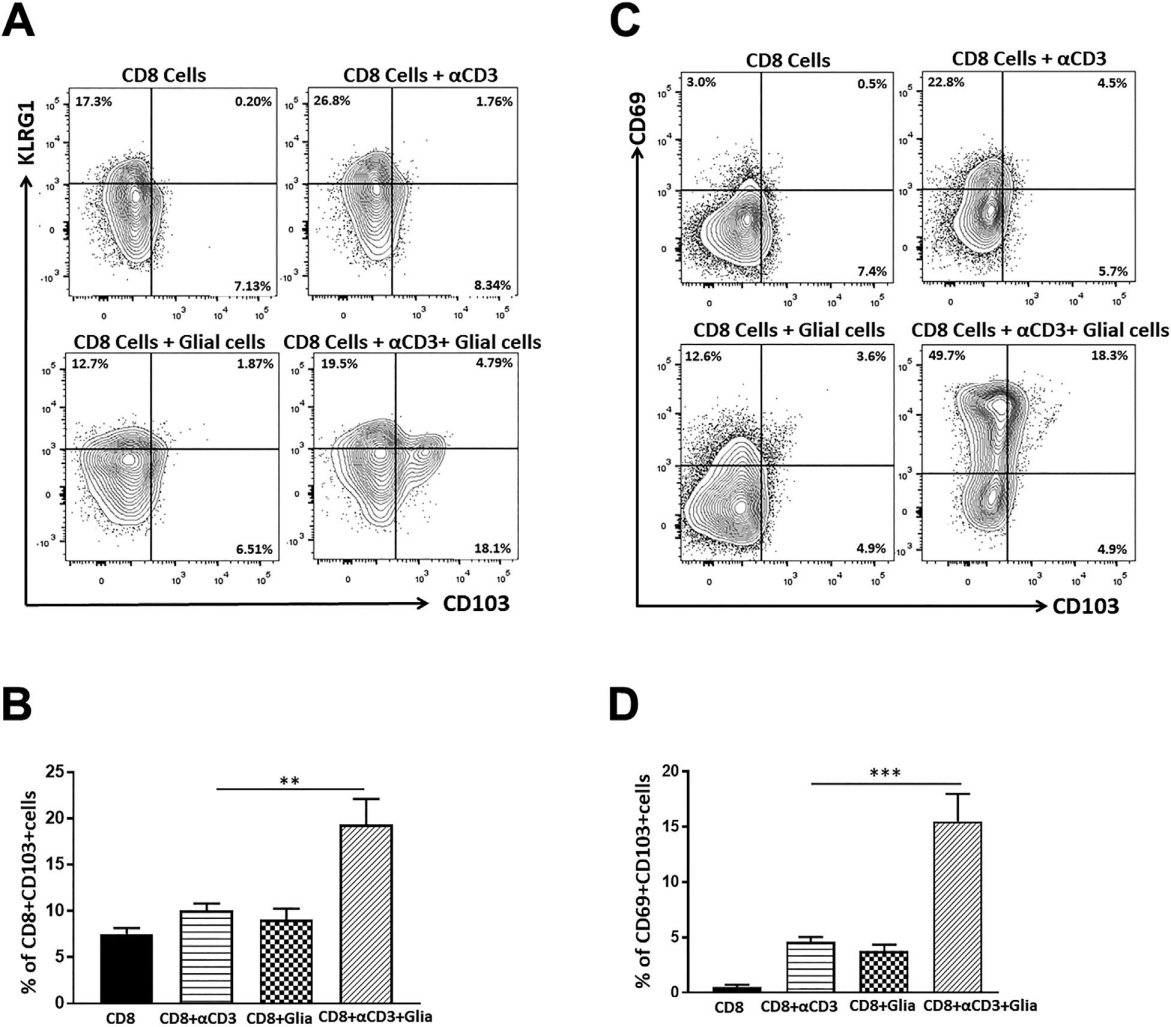
Increased expression of CD103 as well as co‐expression of CD103 and CD69 on CD8^+^ T‐cells in the presence of glia. CD8^+^ T‐cells were isolated from spleens of uninfected C57BL/6 mice using a negative selection kit. CD8^+^ T‐cells were either left unstimulated or stimulated with anti‐CD3 Ab for 1 h prior to transfer into co‐culture with mixed glial cells. CD8^+^ T‐cells were added at a 10:1 CD8: glial cell ratio. Cells were collected at 48 h of culture and analyzed for the expression of KLRG1 and CD103 on CD8^+^ T‐cells. (A) Flow cytometry contour plots show the expression of KLRG1 and CD103 under the indicated culture conditions. (B) Data are representative of three separate experiments. **p < 0.01. (C) Representative contour plots show the co‐expressing CD69^+^CD103^+^ population from gated CD8^+^ T‐cells. (D) Pooled data show percentage (mean ± SD) co‐expression of CD69^+^CD103^+^ cells on CD8^+^ T‐cells from three separate experiments. ***p < 0.001.

We went on to determine if the presence of glial cells promotes expression of CD103^+^ on CD8^+^ T‐cells. In these experiments, we co‐cultured CD8^+^ T‐cells with a mixed culture of primary murine glial cells consisting of approximately 75% astrocytes and 25% microglia. The CD8^+^ T‐cells were first pretreated with anti‐CD3 Ab for 1 h before being co‐cultured with the mixed glial cell culture. We found that anti‐CD3‐stimulated CD8^+^ cells in co‐cultures presented a significant increase in CD103 expression (19.3 ± 2.7%) when compared to anti‐CD3‐stimulated CD8^+^ T‐cells alone (10.0 ± 0.7%) or unstimulated CD8^+^ T‐cells with mixed glial cells (9.0 ± 1.1%). Unstimulated CD8^+^ T‐cells alone also expressed low levels of CD103 (7.5 ± 0.6%), (Fig. 2A, B). Furthermore, analysis of killer like lectin receptor 1 (KLRG1, a marker of SLEC) in our co‐culture studies showed that expression of KLRG1 increased when CD8^+^ T‐cells were stimulated with anti‐CD3 Abs in absence of mixed glial cells (28.3 ± 2.6%), however, its expression was reduced in the presence of glia (16.9 ± 4.0%).

T_RM_ cells are phenotypically distinct and that both CD103 and CD69 are required for optimal formation and survival in various tissues like skin and lung 41. In this study, we examined whether CD8^+^ T‐cells upregulated co‐expression of CD69 and CD103 when co‐cultured with mixed glial cells. Co‐cultures of anti‐CD3 Ab‐stimulated CD8^+^ T‐cells with mixed glial cells resulted in increased co‐expression of CD69 and CD103 on CD8^+^ T‐cells (15.4 ± 2.4%). In contrast, CD8^+^ T‐cells from co‐cultures of unstimulated CD8^+^ T‐cells with mixed glia showed lower co‐expression of CD69 and CD103 (3.7 ± 0.6%), (Fig. 2C, D). Higher proportions of CD69^+^ CD8^+^ T‐cells were observed in the presence of mixed glia (55.1 ± 10.3%), when compared to anti‐CD3 treated CD8^+^ T‐cells in the absence of glial cells (23.4 ± 2.5%), (Fig. 2C).

### Loss of PD‐1 resulted in decreased expression of CD103 on CD8^+^ T‐cells

**Figure 3:**
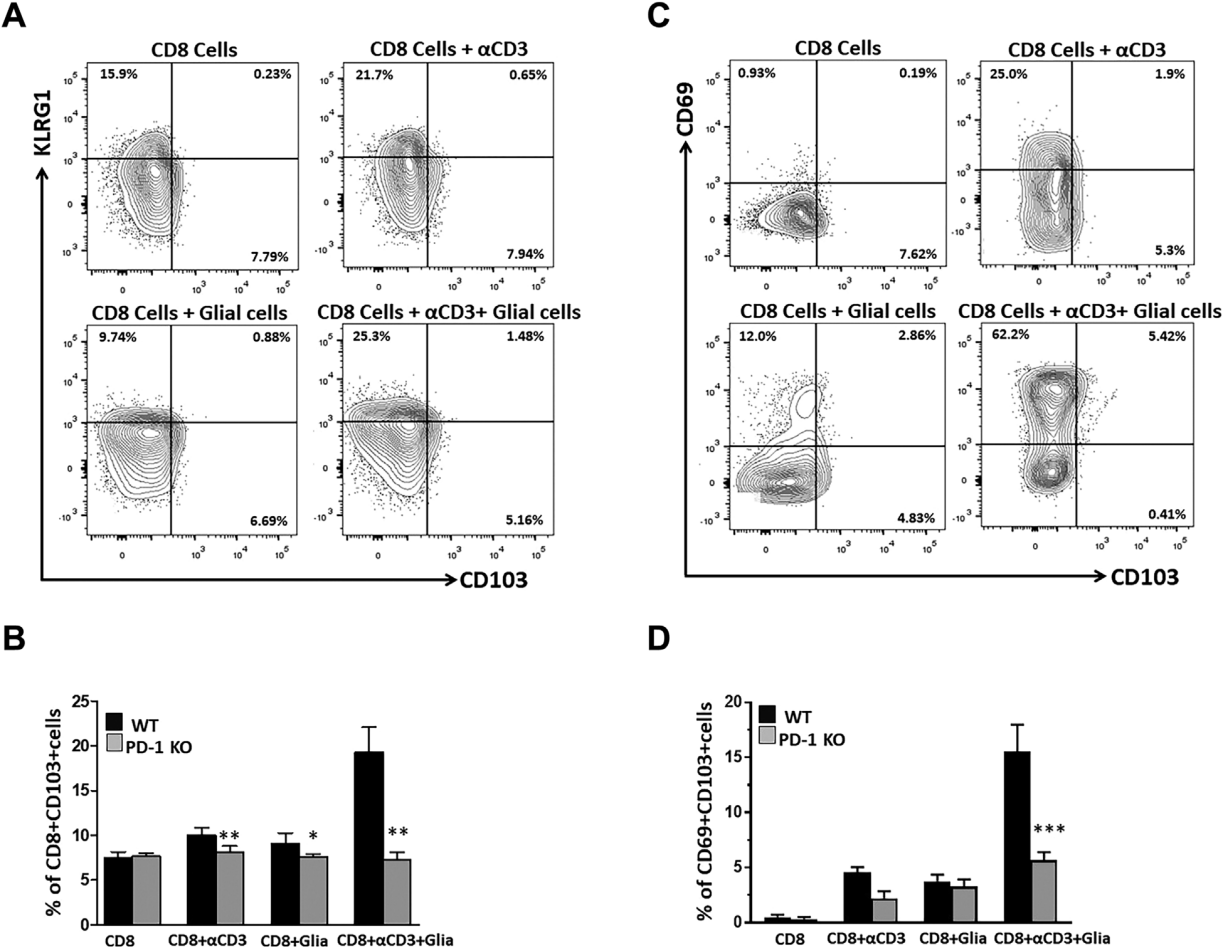
Loss of PD‐1 resulted in decreased expression of CD103 as well as reduced CD69^+^CD103^+^ co‐expression on CD8^+^ T‐cells. CD8^+^ T‐cells from uninfected PD‐1 KO mice were either left unstimulated or stimulated with anti‐CD3 Ab and co‐cultured with mixed glial cells. Cells were collected at 48 h of culture and analyzed for the expression of CD103 and co‐expression of CD69^+^CD103^+^ on gated CD8^+^ T‐cells. (A) Representative contour plots show analysis of KLRG1 and CD103 expression on gated CD8^+^ T‐cells obtained from PD‐1 KO mice. (B) Data show frequency of CD103 expressing CD8^+^ T‐cells obtained from both WT (presented in Figure 2) and PD‐1 KO animals. (C) Flow cytometry plot shows co‐expression of CD69 and CD103 on gated CD8^+^ T‐cells from PD‐1 KO mice. (D) Pooled data present the frequency (mean ± SD) of co‐expressed CD69 and CD103 cells among different groups of mice under the indicated culture conditions. Data are representative of three separate experiments. *p < 0.05 **p < 0.01 ***p < 0.001.

The direct inhibitory effect of glial cell PD‐L1 on CD8^+^ T‐cell activation has been well‐established 1, 8; and we observed that reactive mixed glial cells correlate with increased co‐expression CD103 and CD69. To further, investigate if glial cells modulate CD103 expression through PD‐1: PD‐L1 signaling, we performed in vitro experiments where unstimulated CD8^+^ T‐cells or anti‐CD3 Ab‐stimulated CD8^+^ T‐cells from PD‐1 KO animals were co‐cultured with mixed glia. In these studies, co‐cultures of anti‐CD3, stimulated CD8^+^ T‐cells with mixed glial cells presented a significant decrease in the expression of CD103 on CD8^+^ T‐cells obtained from PD‐1 KO animals (7.2 ± 0.8%), (Fig. 3A, B) when compared to CD103 expression on CD8^+^ T‐cells from WT animals (19.3 ± 2.7%), (Figs. 2D and 3B). Additionally, we also evaluated the co‐expression of CD69 and CD103 on CD8^+^ T‐cells, as we did among WT animals. Co‐cultures of stimulated CD8^+^ T‐cells with mixed glial cells resulted in significantly decreased co‐expression of CD69 and CD103 on cells from PD‐1 KO animals (5.6 ± 0.7%), when compared to its expression on CD8^+^ T‐cells from WT animals (15.4 ± 2.4%), (Fig. 3C, D). However, a higher proportion of CD69^+^ cells was observed using T‐cells from PD‐1 KO animals in the presence of mixed glial cells (59.5 ± 6.9%), when compared to treated CD8^+^ T‐cells in absence of glia (20 ± 6.9%).

### Decreased expression of CD127 on CD69^+^CD8^+^ T‐cells in the absence of PD‐1

**Figure 4:**
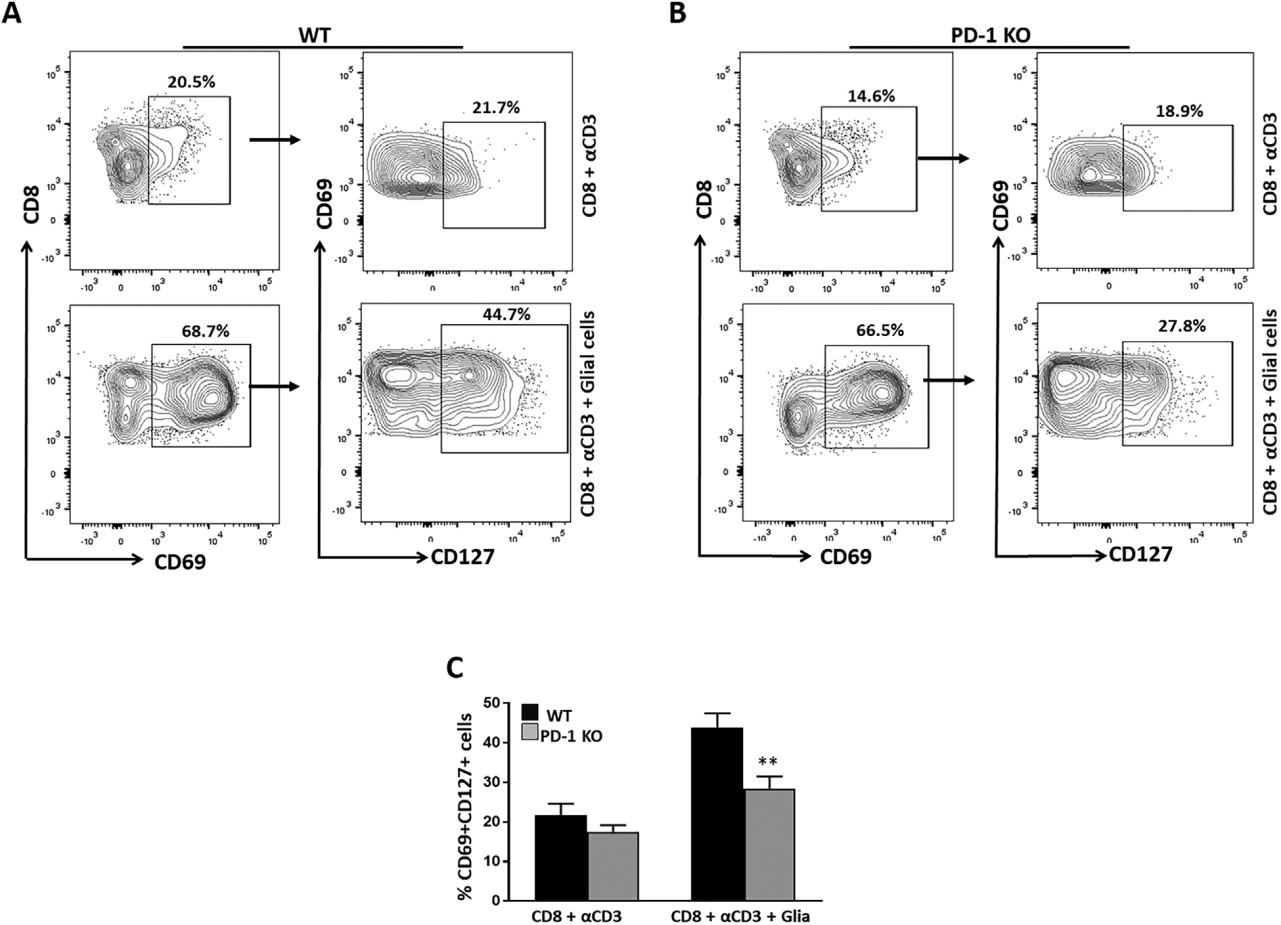
Decreased expression of CD127 on CD69^+^CD8^+^ T‐cells in absence of PD‐1. (A) Representative contour plots show expression of CD127 on CD69^+^gated CD8^+^ T‐cells obtained from WT animals. (B) Contour plot shows expression of CD127 on CD69^+^ cells gated on CD8^+^ T‐cells from PD‐1 KO mice. (C) Pooled data from three separate experiments present the percentage (mean ± SD) of CD127 expression on CD69^+^ CD8 T‐cells among different groups of animals under the indicated culture conditions. **p < 0.01.

Following our observation that stimulated CD8^+^ T‐cells cultured in the presence of mixed glial cells dramatically increased CD69 expression, we further evaluated whether CD69^+^CD8^+^ T‐cells were activated or whether they possessed a memory phenotype. To identify memory precursor effector cells (MPEC), which further give rise to T_RM_ cells, we analyzed the expression of CD127 on the CD69^+^ CD8^+^ T‐cells. A significant population of CD69^+^CD8^+^ T‐cells was found to express CD127 when co‐cultured in the presence of mixed glia. Inversely, anti‐CD3 stimulated CD8^+^ T‐cells alone (i.e., without glia) displayed lower expression of CD127 on CD69^+^CD8^+^ T‐cells (Fig. 4A). Furthermore, to investigate the role glia in promoting memory T‐cell generation, we performed a similar set of experiments using CD8^+^ T‐cells from PD‐1 KO animals, and analyzed expression of CD127 on CD69^+^CD8^+^ T‐cells. In these studies, CD127 expression was found to be significantly reduced on CD69^+^CD8^+^ T‐cells from PD‐1 KO animals in co‐culture with mixed glial cells, when compared to WT (Fig. 4B, C).

### CD69^+^CD103^+^CD8^+^ T‐cells showed reduced T‐bet expression in the presence of glia

**Figure 5:**
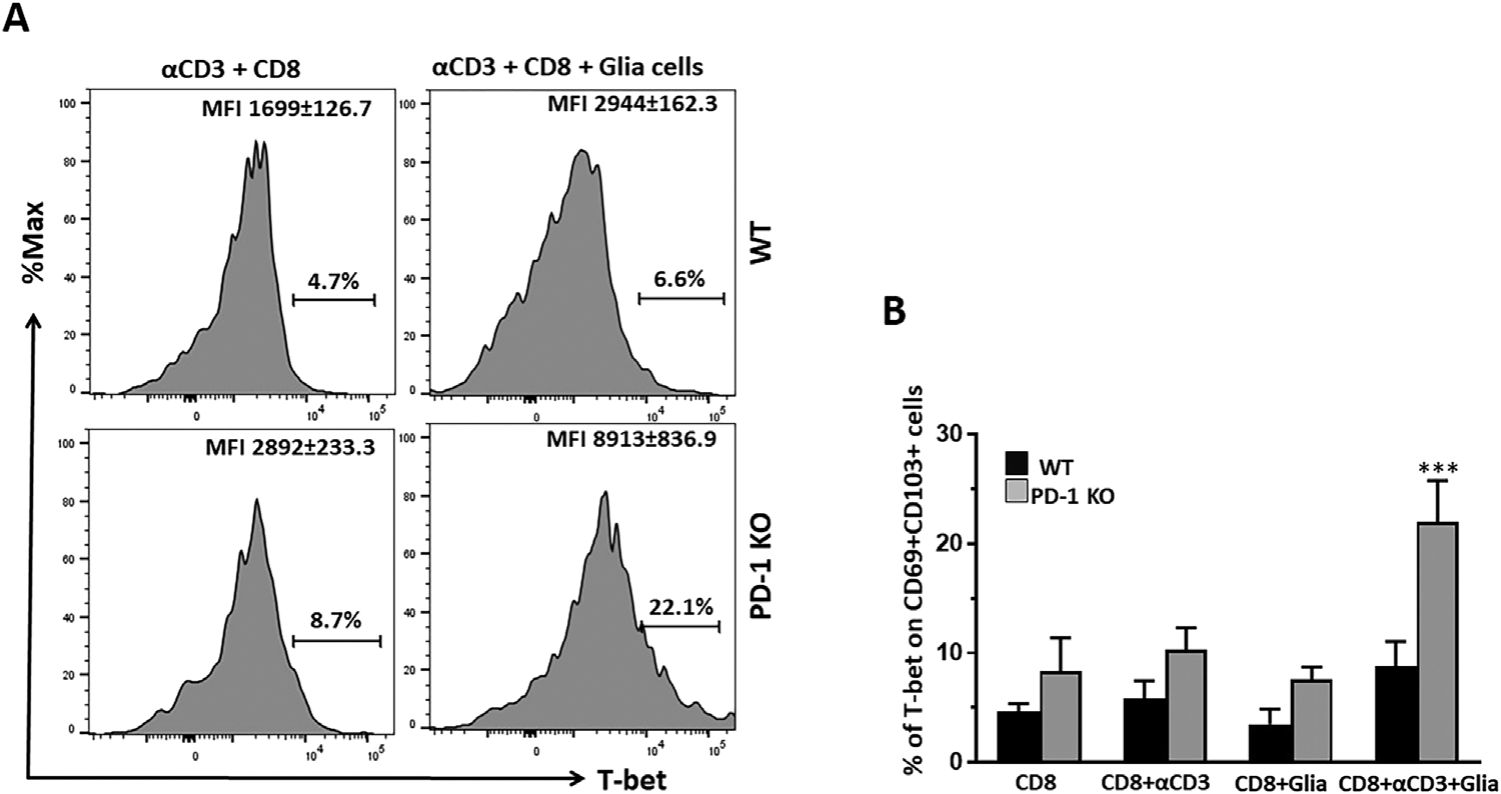
CD69^+^CD103^+^CD8^+^ T‐cells in presence of reactive glia show reduced T‐bet expression. The transcription factor T‐bet was assessed on the CD69^+^CD103^+^ gated CD8^+^ T‐cell population. (A) Histogram plots show expression of T‐bet (as mean fluorescence intensity) on CD69^+^CD103^+^ cells from WT and PD‐1 KO animals in both the presence and absence of reactive glia. (B) Data are presented as (mean ± SD) percentage of T‐bet expression in CD69^+^CD103^+^ cells, on the gated CD8^+^ T‐cell population among WT and PD‐1 KO animals under the indicated culture conditions. ***p < 0.001.

It is increasingly evident that the T‐box transcription factors: T‐bet, homolog of Blimp1 in T‐cells (Hobit), and Eomesodermin (Eomes) tightly regulate the process of memory formation 27, 42, 43, 44. To determine the role of glia in promoting T‐cell memory development, we next examined the expression patterns of T‐bet under various culture conditions by flow cytometry. In these studies, co‐cultures of anti‐CD3‐stimulated CD8^+^ T‐cells and mixed glial cells showed significantly reduced expression of T‐bet (8.8 ± 2.2%) when compared to anti‐CD3 stimulated CD8^+^ T‐cells from PD‐1 KO animals under the same culture conditions (22 ± 3.7%), (Fig. 5A, B). In contrast to the T‐bet phenotype, we found that expression of Eomes increased when anti‐CD3‐stimulated CD8^+^ T‐cells were co‐cultured with glial cells (28.7 ± 1.7%), compared to its expression in the absence of glia (8.8 ± 2.1%). This phenotype was consistent with CD8^+^ T‐cells from PD‐1 KO animals (21.2 ± 4.7% vs. 6.9 ± 3.2% with and without glial cells, respectively). Thus, although Eomes expression was clearly elevated in presence of glial cells, no difference was noted between WT and KO animals (Fig. S1). Taken together, our results suggests that reduced expression of T‐bet on CD69^+^CD103^+^CD8^+^ T‐cells could bias these cells towards generation of memory in the presence of reactive glia.

### Glial cells promote memory development through the PD‐1: PD‐L1 pathway

**Figure 6:**
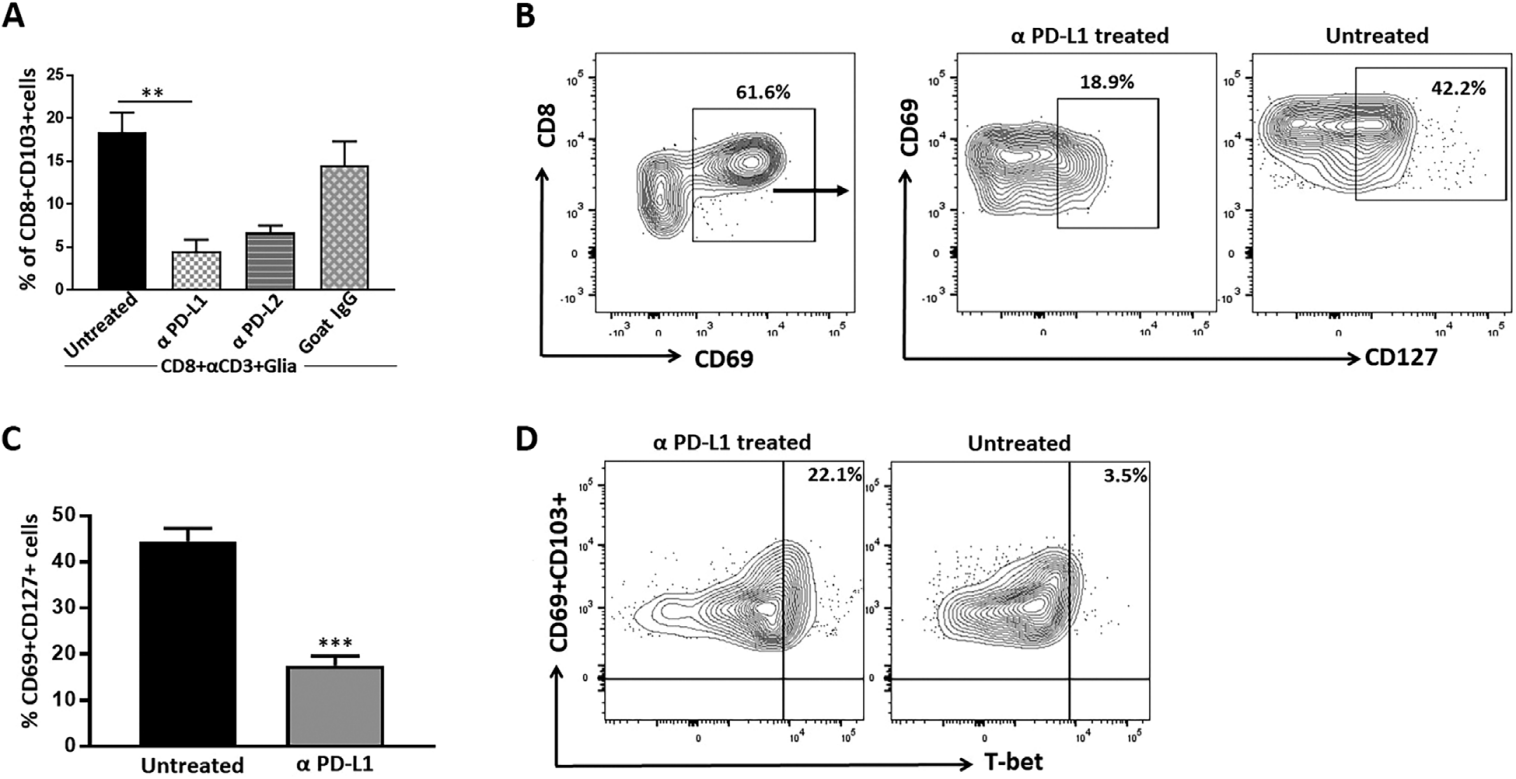
Reactive glia promote memory T‐cell development through the PD‐1: PD‐L1 pathway. Prior to CD8^+^ T‐cell addition, mixed glial cells either left untreated (control) or treated for 2 h with α‐PD‐L1 or α‐PD‐L2 neutralizing Ab. Treatment with rat IgG2a was used as an isotype Ab control. (A) Data show expression of CD103 on CD8^+^ T‐cells from treated and untreated groups from three separate experiments. **p ≤ 0.01. (B) Contour plot represents expression of CD127 on CD69 gated CD8^+^ T‐cells from groups with and without treatment. (C) Pooled data from three separate experiments present the percentage (mean ± SD) of CD127 expression on CD69^+^ CD8 T‐cells among treated and untreated groups ***p < 0.001. (D) Flow cytometric contour plots display expression of T‐bet on CD69^+^CD103^+^ cells among untreated as well as neutralizing Ab‐treated groups.

To further define the functional role of PD‐L1 expression on glial cells in generation of T‐cell memory, we performed in vitro experiments to model its role within the brains of post‐encephalitic mice. Since both activated microglia and astrocytes upregulate expression of PD‐L1 1, we first determined the effects of blocking this negative checkpoint inhibitory pathway. Likewise, blocking of PD‐L1 on mixed glial cells using anti‐PD‐L1 neutralizing Ab, added 1 h prior to addition anti‐CD3 stimulated CD8^+^ T‐cells, resulted in significantly reduced expression of CD103 on CD8^+^ T‐cells when compared to untreated and IgG2a‐treated control (Fig. 6A). Blockade of PD‐L2 on mixed glial cells also resulted in decreased expression of CD103 on CD8^+^ T‐cells; however, this reduction was not as pronounced as PD‐L1 blockade (Fig. 6A). Additionally, to further confirm the role of glial cell PD‐L1 in generation of memory, we evaluated CD127 expression on CD69^+^ CD8^+^ T‐cells. Interestingly, co‐cultures of mixed glia treated with anti‐PD‐L1 neutralizing Ab prior to the addition of anti‐CD3 Ab‐stimulated CD8^+^ T‐cells, also presented significantly lower expression of CD127 on CD69^+^ CD8^+^ T‐cells (17.5 ± 2.0%) when compared to untreated controls (44.4 ± 2.8%), (Fig. 6B,C). These findings were similar to results obtained using PD‐1 KO animals (Fig. 4B, C). Furthermore, we went on to evaluate T‐bet expression on CD69^+^CD103^+^ CD8^+^ T‐cells when anti‐CD3‐treated CD8^+^ T‐cells were in co‐culture with mixed glial cells pretreated with anti‐PD‐L1 neutralizing Ab. Interestingly, we observed elevated T‐bet expression on CD69^+^CD103^+^ CD8^+^ T‐cells with blocking of PD‐L1 (20.5 ± 1.5%) when compared to IgG2a‐treated isotype controls (5.6 ± 1.6%), (Fig. 6D). These results were consistent with findings obtained using PD‐1 KO animals, thus confirming the role of reactive glial cells in generating CD8^+^ T‐cells possessing a memory phenotype through PD‐1: PD‐L1 signaling.

## Discussion

Using an animal model of chronic brain infection following MCMV‐induced encephalitis, we previously demonstrated that activated resident glial cells regulate antiviral T‐cell responses through the PD‐1: PD‐L1 pathway. To manage the deleterious consequences of long‐term persistent neuroinflammation, glial cells upregulate PD‐L1 in response to IFN‐γ produced by infiltrating T‐cells 1. Additionally, more recent work has shown that infiltrating T‐cells which persist long‐term within in the brain acquire a T_RM_ phenotype, and PD‐1: PD‐L1 signaling contributes to the development of this phenotype 10, 45. T_RM_ cells are phenotypically distinct from other memory cell types and afford superior protection against reinfection. Work presented here identifies the role of glial cells in bT_RM_ development through PD‐1: PD‐L1 signaling. Within the brain, T_RM_ cells have largely been defined by co‐expression of CD69 and CD103 46, 47.

A significantly reduced frequency of MCMV‐specific bT_RM_ was seen within the brain of PD‐L1 KO animals when compared to WT animals at d 30 p.i. The reduced accumulation of tetramer‐specific bT_RM_ cells may be related to failed survival of these cells in the absence of PD‐L1, as demonstrated previously using Bcl2 expression 10.

Like T_RM_ cells in other non‐lymphoid organs, bT_RM_ cells are defined by their ability to persist and show immediate effector function upon antigen re‐challenge. Findings presented here demonstrate that antigen‐specific bT_RM_ cells drive potent immune responses upon ex vivo peptide stimulation. We assessed the immunological function of bT_RM_ cells in MCMV‐infected animals in terms of their ability to produce IFN‐γ. A high percentage of antigen specific bT_RM_ cells produce IFN‐γ upon re‐stimulation with epitope‐specific peptides among WT animals, thus indicating potent effector function of bT_RM_. Our finding is in line with other work which demonstrates that the effector function of bT_RM_ cells produced IFN‐γ both in situ and in response to ex vivo peptide stimulation 48. It has been reported that treating T_RM_ cells with PD‐L1 blocking Abs results in increased cytokine production 47, 48. Our previous data report that blockade of the PD‐1: PD‐L1 pathway in both microglia and astrocyte: CD8 T‐cell co‐cultures resulted in increased IFN‐γ and IL‐2 production 1. However, here we first gated for tetramer‐specific CD103+ CD8+ T‐cells from WT and PD‐L1 KO animals at 30 dpi; and then looked for IFN‐γ production by only the tetramer‐specific bTRM cells during recall responses (i.e., not the total CD8+ T‐cell response). Additionally, we found the population of CD103^‐^ CD8^+^ T‐cells observed in PD‐L1 KO animals produced more IFN‐γ (8.4 ± 1.2%) than those of WT animals (1.0 ± 0.19%) upon ex vivo stimulation (Fig. S2). Similar findings have been reported using Theiler's murine encephalomyelitis virus (TMEV), where the authors noted that depletion of PD‐L1 resulted in an increased population of CD103^−^ CD8^+^ T‐cells that produced IFN‐γ, which provides additional evidence that IFN‐γ production by CD103^−^ CD8^+^ T‐cells suppressed the accumulation of T_RM_ in PD‐L1 KO animals 45.

Resident microglial cells exhibit properties which are similar to macrophages that infiltrate the brain in response to infection or injury. Evidence suggests that activated microglia display APC function and upregulate receptors necessary to interact with infiltrating T‐cells, thereby contributing to immune surveillance and homeostasis in the brain 3. In vitro studies from our laboratory demonstrated that both microglia and astrocytes upregulated MHC I and II, as well as PD‐L1 in response to IFN‐γ produced by anti‐CD3 Ab‐stimulated CD8^+^ T‐cells. It is well‐established that microglia possess a number of mechanisms to limit CNS inflammation and regulate immune responses in various disease settings 1, 49. In this study, we identified the role of glial cells to promote the development of bT_RM_ cells. Our data indicates that glial cells have the potential to promote expansion of CD69^+^CD103^+^ CD8^+^ T‐cells when anti‐CD3 Ab‐stimulated CD8^+^ T‐cells were in co‐culture. However, reduced expression of CD69^+^CD103^+^ CD8^+^ T‐cells from PD‐1 KO animals in identical co‐culture conditions indicates the involvement of PD‐1: PD‐L1 signaling. A role for microglia in modulating immune cells has been demonstrated in various studies 35, 49, 50. It has been demonstrated that microglia promote effector T‐cells and T‐regulatory (Treg) cell induction in presence of IFN‐γ (Friederike Ebner 2013,). It has also been shown that under in vivo conditions PD‐1 deficiency delayed the switch from an M1 to M2 microglial cell polarization phenotype after spinal cord injury 51.

Our laboratory and others have previously reported the early induction of CD69 expression on brain infiltrating effector T‐cells, as well as the local conversion of infiltrating CD8^+^ T‐cells to CD69^+^CD103^+^ cells within MCMV‐infected brain 9, 10, 35, 46, 52. Other studies report that dendritic cell accumulation in skin epithelium and dermis can provide Ag and type I IFN for CD69 induction in T‐cells in vitro; however, Ag and type I IFN are dispensable for CD69 expression in vivo [17]. Previous studies report that expression of CD69 is not just a marker of activation, but rather is also an important immune regulator. In this study, we observed that reactive glia promoted heightened expression of CD69 on CD8^+^ T‐cells amongst both WT and PD‐1 KO animals. Thus, further phenotypic analysis of CD69 was critical. Different subsets of antiviral CD8^+^ T‐cells emerge following infection. Expression of the IL‐7 receptor α chain (CD127) expression differentiates CD8^+^ T‐cells into different subsets following infection 10, 53, 54, 55. In mice infected with lymphocytic choriomeningitis virus (LCMV), it has been demonstrated that IL‐7Rα^+^ effector CD8^+^ T‐cells expressed higher levels of Bcl‐2 than their IL‐7Rα^‐^ counterparts, suggesting IL‐7Rα^+^ effector cells survive and develop into long‐lived memory CD8^+^ T‐cells 53, 56. Surprisingly, in our study CD69^+^CD8^+^ T‐cells displayed heightened expression of CD127 (43.8 ± 3.6%) in the presence of activated glial cells, whereas anti‐CD3 Ab‐stimulated CD8^+^ T‐cells in absence of glia showed reduced expression of CD127 (21.8 ± 2.8%). Similarly, PD‐1 deficient CD69^+^ CD8^+^ T‐cells resulted in significantly reduced expression of CD127 (28.5 ± 2.9%), further indicating the role of activated glia in promotion of long‐lived memory cells through the PD‐1: PD‐L1 pathway.

Previous studies have implicated involvement of the transcription factors T‐bet and Eomes in acquisition of CD8^+^ T‐cell effector function and the development of memory CD8^+^ T‐cells 15, 27, 43. Therefore, we analyzed the expression of both T‐bet and Eomes in CD69^+^CD103^+^ CD8^+^ T‐cells to further confirm that glial cells promote memory generation. Additional analysis revealed that expression of T‐bet was higher in CD69^+^CD103^+^ CD8^+^ T‐cells from PD‐1 KO animals, indicating more cells possessing an effector phenotype; whereas cells from WT animals showed decreased T‐bet indicative of more long‐lived cells. Interestingly, PD‐L1 blocking also resulted in increased expression of T‐bet, further suggesting the involvement of reactive glia through PD‐1: PD‐L1 signaling. These findings were in line with other studies where T‐bet expression was found to be highest in short‐lived effector cells (SLEC), 23, 29. Additionally, our data shows that lack of PD‐1: PD‐L1 signaling did not significantly compromise the expression of Eomes (Fig. S1). However, expression of Eomes on CD8^+^ T‐cells increased in the presence of mixed glia. Our observation was consistent with Joshi et al., and others, who reported that Eomes expression is upregulated in long‐lived memory cells in vivo 29, 57, 58. In contrast to these findings, it has also been demonstrated that brain CD103^+^ T_RM_ cells express low levels of the transcription factors T‐cell factor 1 (Tcf‐1) and Eomes. Tcf‐1 deficient cells were associated with low levels of Eomes and were not found to be critical in the lodgment of memory T‐cells within peripheral tissues 46. Addition data of also revealed that brain T_RM_ cells failed to undergo recall expansion when dissociated from tissue, thus indicating differences depending upon the microenvironment of the T_RM_ cells. In addition, T‐bet was downregulated, whereas Eomes expression was completely lost in skin T_RM_ cells induced by herpes simplex virus (HSV)‐1 infection. Moreover, forced expression of either T‐bet or Eomes prevented formation of T_RM_ cells 25, 26. These findings illustrate that signals which trigger T_RM_ formation differ in diverse microenvironments.

## Conclusions

Taken together our findings identify the role of activated glial cells in promoting the development of bT_RM_ cells through interaction of the PD‐1: PD‐L1 pathway. Importantly our data demonstrate previously unidentified interactions between glia and CD8^+^ T‐cells in the generation of long‐lived memory cells. Findings from PD‐1 KO animals and PD‐L1 blocking studies addressed the involvement of activated glia in shaping infiltrating CD8^+^ T‐cells as indicated by the reduced expression of CD127 on CD69^+^CD8^+^ T‐cells among PD‐1 KO animals. Thus, our findings enhance understanding regarding how the brain microenvironment shapes infiltrating CD8^+^ T‐cells to generate bT_RM_.

## Conflict of Interest

None declared.

## Supporting information

Additional supporting information may be found in the online version of this article at the publisher's web‐site.

**Figure S1**. Expression of Eomes on CD69^+^CD103^+^CD8^+^ T‐cells from WT and PD‐1 KO animals. CD8^+^ T‐cells from uninfected WT and PD‐1 KO mice were either left unstimulated or stimulated with anti‐CD3 Ab and were co‐cultured with mixed glial cells. CD8^+^ T‐cells were added at a 10:1 CD8: glial cell ratio. Cells were collected at 48 h of culture and analyzed for the expression of Eomes on CD69^+^CD103^+^ gated CD8^+^ T‐cells (A). Gating strategy used for analysis of *in vitro* expression. (B) Representative contour plots show the percentage of Eomes expression on CD69^+^CD103^+^ gated CD8^+^ T‐cells obtained from WT and PD‐1 KO under various culture conditions.

**Figure S2**. IFN‐γ production by CD103^‐^CD8^+^ T‐cells from WT and PD‐1 KO animals. (A) Flow cytometric analysis of brain mononuclear cells obtained from MCMV‐infected WT and PD‐L1 KO animals at 30 d post infection represents reduced CD103 expression in PD‐L1 KO compared to WT animals. (B) CNS‐derived lymphocytes were gated on CD103^−^ CD8^+^ T‐cells and representative contour plots show IFN‐γ production by the CD103^−^ population of CD8^+^ T‐cells from WT and PD‐L1 KO mice at 30dpi.
