# Supplementary figures and images for: Reactive glia promote development of CD103+CD69+ CD8+ T‐cells through programmed cell death‐ligand 1 (PD‐L1)
Source: Immun Inflamm Dis. 2018 Mar 30;6(2):332–44. doi: 10.1002/iid3.221 (PMC5946148; doi:10.1002/iid3.221)

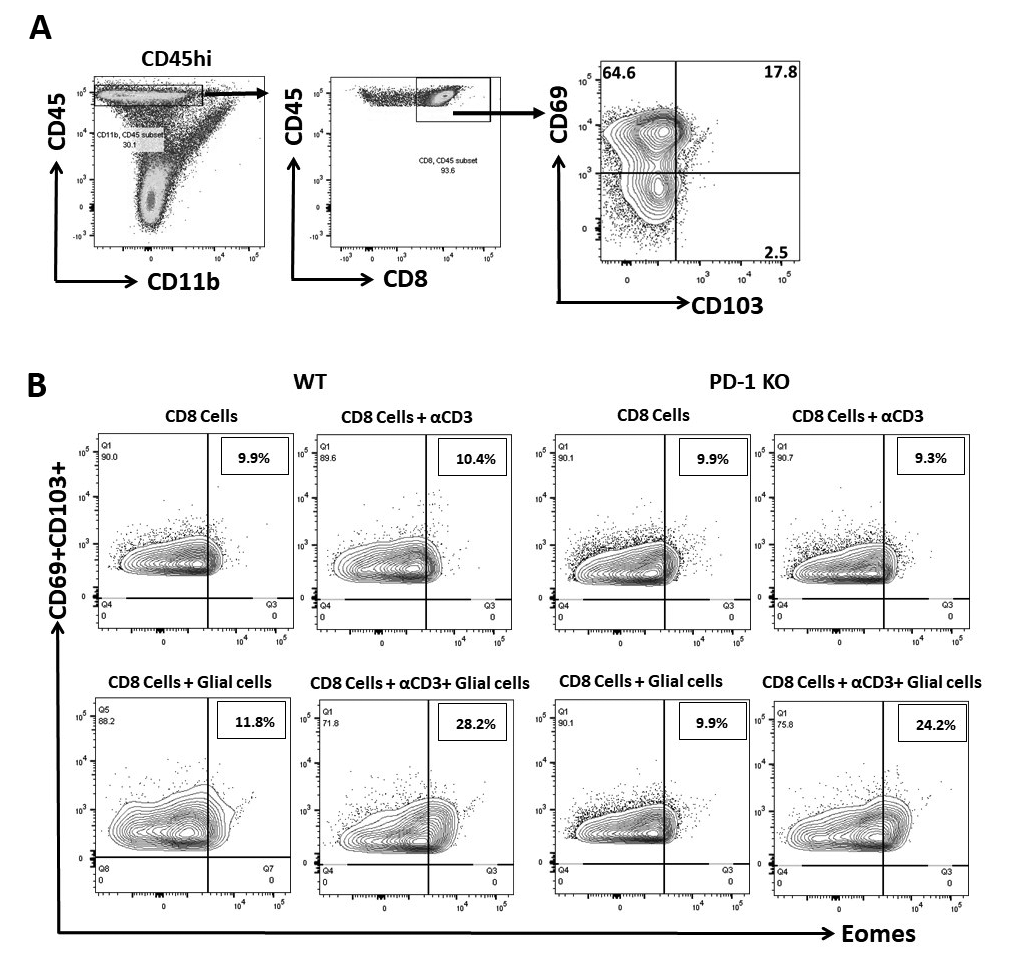

Supplement: Supplementary file 1 — Figure S1. Expression of Eomes on CD69+CD103+CD8+ T‐cells from WT and PD‐1 KO animals. CD8+ T‐cells from uninfected WT and PD‐1 KO mice were either left unstimulated or stimulated with anti‐CD3 Ab and were co‐cultured with mixed glial cells. CD8+ T‐cells were added at a 10:1 CD8: glial cell ratio. Cells were collected at 48 h of culture and analyzed for the expression of Eomes on CD69+CD103+ gated CD8+ T‐cells (A). Gating strategy used for analysis of in vitro expression. (B) Representative contour plots show the percentage of Eomes expression on CD69+CD103+ gated CD8+ T‐cells obtained from WT and PD‐1 KO under various culture conditions. [file IID3-6-332-s001.tif]

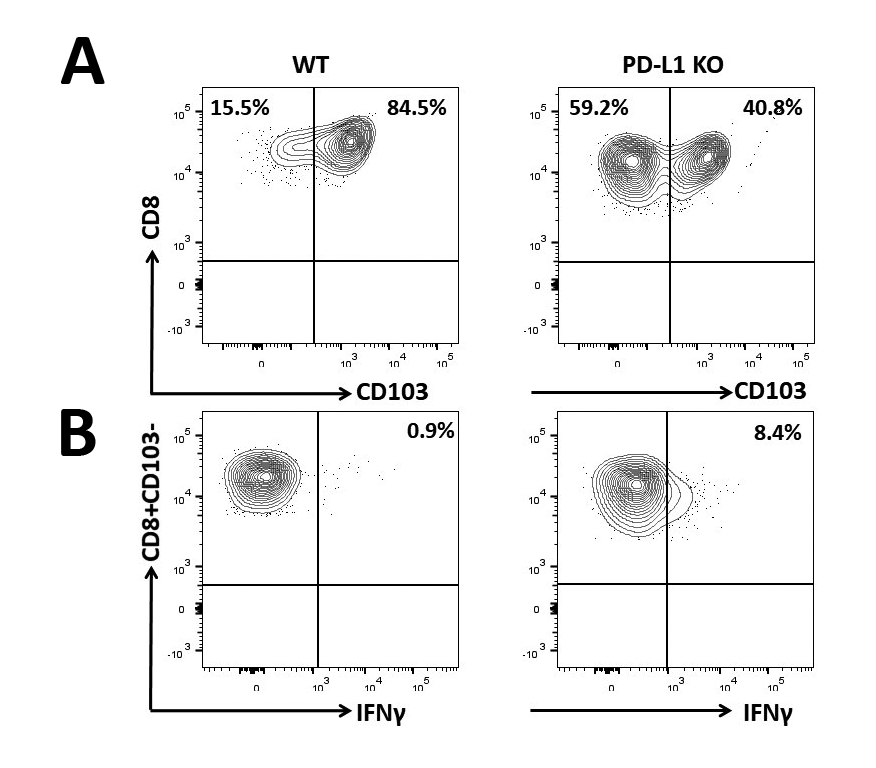

Supplement: Supplementary file 2 — Figure S2. IFN‐γ production by CD103‐CD8+ T‐cells from WT and PD‐1 KO animals. (A) Flow cytometric analysis of brain mononuclear cells obtained from MCMV‐infected WT and PD‐L1 KO animals at 30 d post infection represents reduced CD103 expression in PD‐L1 KO compared to WT animals. (B) CNS‐derived lymphocytes were gated on CD103− CD8+ T‐cells and representative contour plots show IFN‐γ production by the CD103− population of CD8+ T‐cells from WT and PD‐L1 KO mice at 30dpi. [file IID3-6-332-s002.tif]
